# Magnetotransport signatures of antiferromagnetism coexisting with charge order in the trilayer cuprate HgBa_2_Ca_2_Cu_3_O_8+*δ*_

**DOI:** 10.1038/s41467-022-29134-6

**Published:** 2022-03-23

**Authors:** V. Oliviero, S. Benhabib, I. Gilmutdinov, B. Vignolle, L. Drigo, M. Massoudzadegan, M. Leroux, G. L. J. A. Rikken, A. Forget, D. Colson, D. Vignolles, C. Proust

**Affiliations:** 1 LNCMI-EMFL, CNRS UPR3228, Univ. Grenoble Alpes, Univ. Toulouse 3, INSA-T, Toulouse, France; 2 CNRS, Univ. Bordeaux, Bordeaux INP, ICMCB, UMR 5026, F-33600 Pessac, France; 3 Service de Physique de l’Etat Condensé, CEA Saclay (CNRS-URA 2464), 91191 Gif sur Yvette, France; 4 Present Address: Institute of Physics, EPFL, CH-1015 Lausanne, Switzerland; 5 Present Address: GET (UMR5563 CNRS, IRD, Univ. Paul Sabatier, CNES), 31400 Toulouse, France

**Keywords:** Electronic properties and materials, Superconducting properties and materials

## Abstract

Multilayered cuprates possess not only the highest superconducting temperature transition but also offer a unique platform to study disorder-free CuO_2_ planes and the interplay between competing orders with superconductivity. Here, we study the underdoped trilayer cuprate HgBa_2_Ca_2_Cu_3_O_8+*δ*_ and we report quantum oscillation and Hall effect measurements in magnetic field up to 88 T. A careful analysis of the complex spectra of quantum oscillations strongly supports the coexistence of an antiferromagnetic order in the inner plane and a charge order in the outer planes. The presence of an ordered antiferromagnetic metallic state that extends deep in the superconducting phase is a key ingredient that supports magnetically mediated pairing interaction in cuprates.

## Abstract (web summary)

The interplay between superconductivity and competing orders in multi-layered cuprates can shed light on the nature of the superconducting pairing. Here, the authors report on the coexistence of antiferromagnetic and charge orders in different CuO2 planes in a tri-layer cuprate, pointing to a magnetically-mediated mechanism.

## Introduction

The close proximity of antiferromagnetic (AFM) order to an unconventional superconducting phase is a generic feature of strongly correlated superconductors. The coexistence and interplay of AFM order and superconductivity have led to theories based on spin-fluctuation mediated pairing interaction^1^. In cuprate high-temperature superconductors, magnetic interactions are at the heart of the debate for the pairing interaction. Although the parent compounds are antiferromagnetic Mott insulator, the presence of the pseudogap phase in hole-doped cuprates complicates the situation. Indeed, there is a variety of competing orders with superconductivity, such as charge order, stripe order or nematic phase, that nucleate inside the pseudogap^2,3^. The multi-layered cuprates provide a proving ground for studying such multiple phases. They have been thoroughly studied by NMR^4^, ARPES^5–7^ and Raman spectroscopy^8^. The highest superconducting transition temperature (*T*_*c*_) at ambient pressure is observed for three CuO_2_ planes^9,10^, but the microscopic mechanism at the origin of this experimental observation is still under debate^11–14^. One way to understand this problem is to consider the substantial interplane coupling that could stabilize the AFM phase in the underdoped regime, thus boosting AFM fluctuations away from the ordered phase and close to optimal doping. Moreover, the interplane coupling could suppress phase fluctuations and hence increase *T*_*c*_. Another important ingredient of multi-layered cuprates is the symmetry-inequivalent CuO_2_ planes. Indeed, the fact that the inner planes (IPs) are not adjacent to the charge reservoir has two consequences: (i) the inner CuO_2_ planes are protected from out-of-plane disorder and extremely clean^4^, and (ii) the fact that IPs are farther from the charge reservoir layer than outer planes (OPs) induces a charge imbalance between the different planes. This has been demonstrated by NMR measurements in several multi-layered cuprates (for a review, see ref. ^4^) and by ARPES measurements^7^ in optimally doped tri-layer Bi_2_Sr_2_Ca_2_Cu_3_O_10+*δ*_ (Bi2223). Consequently, different competing orders can appear in the IPs and the OPs. And each of these orders could influence the Fermi surface (FS) from which high-*T*_*c*_ superconductivity emerges at optimal doping. Namely, AFM order is known to reconstruct the FS at low doping^5^, and charge order (CO) is also now recognized as a generic property of underdoped cuprates^3^. For instance, in underdoped YBa_2_Cu_3_O_*y*_ (YBCO) and HgBa_2_CuO_6+*δ*_ (Hg1201), the observation of quantum oscillations (QOs) with small frequencies^15,16^ and negative Hall effect^17,18^ are a strong indication of the presence of a small closed electron pocket indicating a FS reconstruction. NMR^19^ and X-ray scattering^20–22^ measurements then found evidence of CO in YBCO and Hg1201. While the exact scenario for the FS reconstruction is still debated^23^, a biaxial CO can indeed lead to an electron pocket in the nodal region of the first Brillouin zone^24^.

Among cuprates, HgBa_2_Ca_2_Cu_3_O_8+*δ*_ (Hg1223) holds the record of the highest superconducting transition temperature at ambient pressure (*T*_*c*_ = 133 K). It is a tri-layer cuprate and the narrow ^63^Cu-NMR linewidth^4,25^ clearly shows that the IP is extremely clean as it is homogeneously doped and screened from out-of-plane disorder by the OPs. In addition, Raman spectroscopy shows the typical signature of CO in optimally and underdoped Hg1223^8^, and NMR measurements performed in an equivalent tri-layer cuprate^4^ suggest the critical doping at which AFM order in the IP disappears corresponds to an average carrier density *p* = 9% (*T*_*c*_ ≈ 80 K). In the following, the doping level of multi-layered cuprates has been estimated from *T*_*c*_ (see Methods) and represents an averaged doping level between the inner and the outer planes. Here, we investigate the transport properties of underdoped Hg1223 in the doping range *p* = 8–8.8% by means of contactless resistance and Hall effect measurements in pulsed fields up to 88 T. We discover quantum oscillations with small frequencies and a Hall coefficient that remains positive down to the lowest temperature, evidencing the presence of small reconstructed pockets of both holes and electrons, which strongly supports the coexistence of AFM and CO but on different CuO_2_ planes, with AFM on the IPs and CO on the OPs. An additional frequency corresponding to magnetic breakdown tunnelling between the inner and outer planes is also observed.

## Results

### Observation and analysis of quantum oscillations

**Fig. 1:**
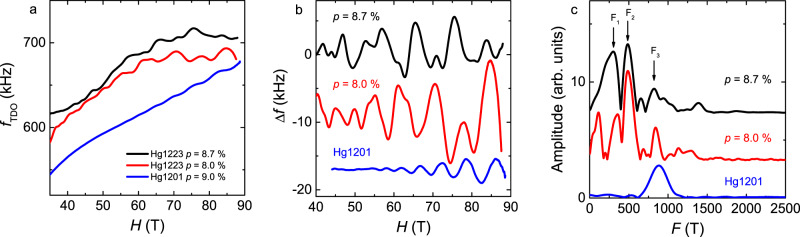
QO data. **a** Field dependence of the TDO frequency after the heterodyne circuit at low temperature in the monolayer Hg1201 (blue line) and in the tri-layer Hg1223 at different doping levels (black and red line). **b** Oscillatory part of the TDO signal after removing a smooth background (spline) from the data shown in panel **a**. **c** Discrete Fourier analysis of the oscillatory part of the TDO signal shown in panel **b**.

Figure 1a shows the variation of the tunnel diode oscillator (TDO) circuit frequency (see Methods) as a function of magnetic field for Hg1201 at *p* = 9% and for two samples of Hg1223 at slightly different doping levels. In the latter, QOs are clearly observed above *H* = 40 T, confirming the high quality of the samples. A smooth background subtraction leads to the oscillatory part of the signal shown in Fig. 1b. While there is obviously only one QO frequency for Hg1201, the QO spectrum of Hg1223 is much more complex and contains several frequencies. This is confirmed by the discrete Fourier transform analysis depicted in Fig. 1c. In Hg1201, the discrete Fourier transform reveals a single frequency *F* = 850 T, in agreement with previous studies^16,26^. For Hg1223, neglecting the low frequencies that can be attributed to imperfect background subtraction (see discussion in the Supplementary Note 2), at least three frequencies can be isolated at *F*_1_ ≈ 350 T, *F*_2_ ≈ 500 T and *F*_3_ ≈ 850 T, where *F*_3_ − *F*_2_ ≈ *F*_1_. Some harmonics and frequency combinations are also present at higher frequencies. The temperature dependences of the QOs are shown in Supplementary Fig. 2. As expected from the Lifshitz-Kosevich theory^27^, the amplitude of QOs decreases as the temperature increases and vanishes above *T* ≈ 10 K.

**Fig. 2:**
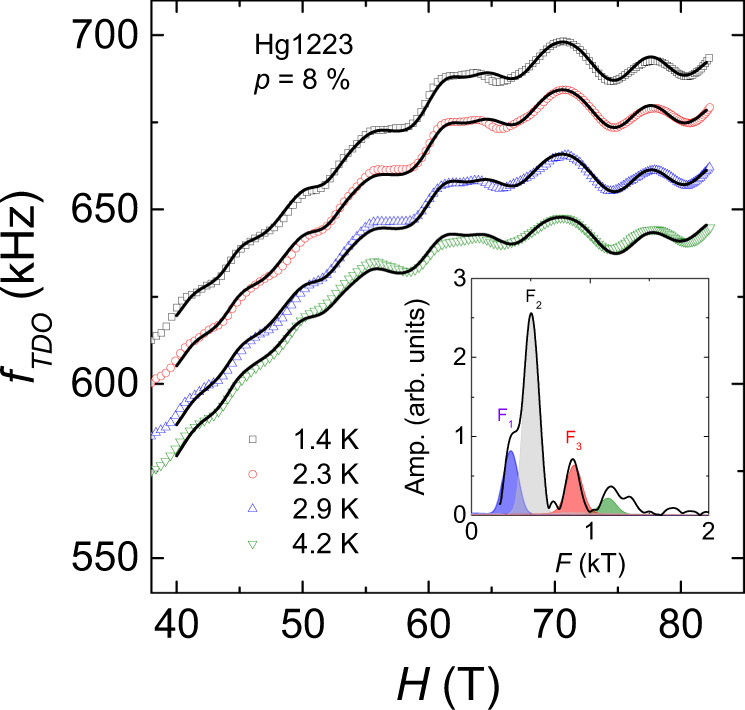
Lifshitz-Kosevich fits. Field dependence of the TDO frequency in Hg1223 (*p* = 8%) at different temperatures (symbols). Solid lines correspond to the fits to the data using the Lifshitz-Kosevich theory plus a polynomial background in the field range 40 ≤ *H* ≤ 83 T and in the temperature range *T* = 1.4–4.2 K (see Supplementary Note 3 for details). The inset shows the Fourier analysis of the oscillatory part of the data at *T* = 1.4 K along with the contribution of *F*_1_ (blue), *F*_2_ (grey) and *F*_3_ (red), respectively. The green component corresponds to a frequency combination, which has been taken into account to improve the fits.

A challenge in analysing these data is that the oscillation frequencies are low and there is a limited field range available. Therefore, the accurate determination of the value of the frequencies is ambiguous, in particular for the nearby frequencies *F*_1_ and *F*_2_. To assert the spectra of QOs, we performed fits to the data at different temperatures using the Lifshitz-Kosevich theory (see Supplementary Note 3 for the detailed procedure of the fit). In order to constrain the fits, we performed simultaneous fits to the dataset at different temperatures (from *T* = 1.4 K to *T* = 4.2 K at *p* = 8%), where all parameters are temperature independent except for the background. Figure 2 shows the raw data for the sample at *p* = 8% (symbols) and solid lines are the simultaneous fits in the temperature range *T* = 1.4–4.2 K (see Supplementary Fig. 3 for the *p* = 8.7% sample from *T* = 0.6 K to *T* = 2.9 K). The value of the frequencies deduced from the fitting procedure at *p* = 8% are *F*_1_ = 331 T, *F*_2_ = 500 T and *F*_3_ = 866 T, in good agreement with the values obtained by discrete Fourier transform at different temperatures (see Table 1). Both analyses confirmed that the oscillatory spectrum is composed of at least three frequencies linked by the relation *F*_3_ − *F*_2_ ≈ *F*_1_.

**Table 1:** Sample family, *T*_*c*_, doping *p* and QO frequency deduced from the discrete Fourier transform analysis.

Family	*T*_*c*_	*p*	*F*_1_	*F*_2_	*F*_3_
Hg1223	64 K	8.0%	330 ± 30 T	500 ± 20 T	850 ± 20 T
Hg1223	74 K	8.7%	335 ± 20 T	500 ± 20 T	850 ± 20 T
Hg1201	74 K	9.7%	×	×	880 T

### Hall effect measurements

**Fig. 3:**
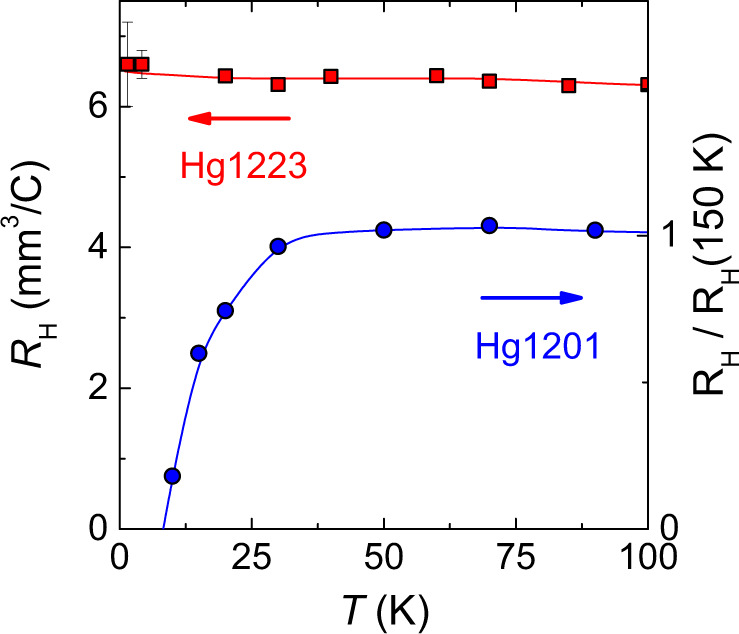
Hall data. Temperature dependence of the normal-state Hall coefficient *R*_*H*_, measured at high fields, in Hg1223 (*p* = 8.8%, red squares) and in Hg1201 (*p* = 8%, blue circles adapted from ref. ^18^). The Hall coefficient changes sign in Hg1201 while it remains positive (i.e. hole-like) down to the lowest temperature in Hg1223. Note that in Hg1223, as *T* → 0, *R*_*H*_ ≈ 6.5 mm^3^/C, corresponding to an effective carrier density *p*_*H*_ ≈ 8%. Error bars represent the noise generated by the 90 T coil (see Supplementary Note 4).

In order to gain more insight into the Fermi surface of underdoped Hg1223, we performed Hall effect measurements up to 88 T at a doping level *p* = 8.8%. Figure 3 shows the temperature dependence of the normal-state Hall coefficient down to *T* = 1.5 K (the isotherms are shown in Supplementary Fig. 4). Remarkably, there is almost no temperature dependence of the Hall coefficient and it remains positive down to the lowest temperatures. This result contrasts with the Hall coefficient in underdoped YBCO^17,28^ and Hg1201^18^, which changes signs and becomes negative at low temperatures. This has been interpreted as the signature of an electron pocket resulting from the FS reconstruction caused by the CO. In the case of underdoped YBCO, the CO is present in both CuO_2_ planes of the bilayer and QO measurements reveal the main frequency *F*_*e*_ = 540 T flanked by two nearby and equally spaced satellites *F*_*e*_ ± 90 T at a doping level *p* ≈ 11%^29,30^. This has been interpreted as magnetic breakdown tunnelling between bilayer-split pockets^30–32^ provided that the mirror symmetry between the planes of the bilayer is broken. In the single-layer Hg1201, only one QO frequency *F* ≈ 850 T has been detected so far^16,26^. The difference in the value of the main frequency between YBCO and Hg1201 is a direct consequence of a different CO wavevector, leading to different sizes of the reconstructed electron pocket.

## Discussion

We now discuss different scenarios to explain our results in underdoped Hg1223. For the sake of simplicity, we treat the inner and the outer planes as independent even if they are coupled by an interlayer tunneling *t*_⊥_. For the Fermi surface reconstruction, we consider only one band within each CuO_2_ plane.

### Scenario (1): Band structure calculations

Local-density-approximation calculations^33^ of the electronic structure of the stoichiometric compound HgBa_2_Ca_2_Cu_3_O_8_ reveal that the Fermi surface consists of three large hole-like tubular CuO_2_ sheets centered on the corner of the Brillouin zone plus a small electron-like Fermi surface located at the anti-node (see Supplementary Fig. 6a). The latter disappears with doping^33^. For a doping *p* ≈ 8%, the Fermi surface of the CuO_2_ sheets corresponds to 1 + *p* holes that translates into a QO frequency *F*_*LDA*_ ≈ 15 kT much larger than the observed frequencies in our study.

### Scenario (2): Charge order in the three CuO_2_ planes

In analogy with underdoped YBCO where a CO is present in both CuO_2_ planes, let us assume that a CO is present in the three CuO_2_ planes of Hg1223 (see a sketch of the scenario in Supplementary Fig. S6b). As the frequencies are not equally spaced, the model of magnetic breakdown tunnelling is inadequate to explain the spectrum of oscillation frequencies in Hg1223. Moreover, the Hall coefficient remains positive down to the lowest temperature, in contrast with underdoped Hg1201 and YBCO.

### Scenario (3): AFM in the inner plane

Another scenario assumes an AFM metallic phase in the IP and a corresponding FS that contains both electron and hole pockets (see a sketch of this scenario in Supplementary Fig. 6c). While there is no direct evidence yet of an AFM order in underdoped Hg1223, such order has been detected by extensive NMR measurements in the IP of the tri-layer cuprate Ba_2_Ca_2_Cu_3_O_6_(F,O)_2_ (O223F) with *T*_*c*_ up to 81 K but not beyond^4,34^. Given the disorder-protected nature of IPs in multi-layered cuprates, let us assume that QOs originate from quasi-particles in the IP with *F*_*hole*_ = *F*_3_ ≈ 850 T and *F*_*electron*_ = *F*_2_ ≈ 500 T. In this scenario, the third frequency *F*_1_ = *F*_3_ − *F*_2_ would correspond to a magnetic breakdown between the hole and the electron pockets in the IP. However, in order to reproduce the size of the orbits corresponding to the observed frequencies *F*_2_ and *F*_3_, the AFM potential used in the calculation is 0.25 eV (see discussion in Supplementary Note 6 and Supplementary Fig. 6c). This value translates to a magnetic breakdown field unattainable, ruling out the possibility to observe magnetic breakdown between the hole and electron pocket. Finally, the presence of an electron pocket at the anti-node is difficult to reconcile with the presence of a pseudogap.

### Scenario (4): AFM in the inner plane and charge order in the outer plane

**Fig. 4:**
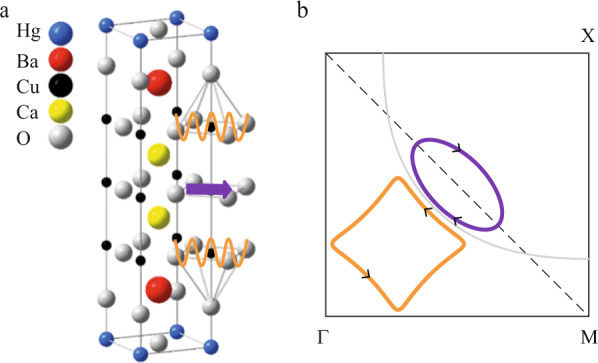
Sketch of the Fermi surface. **a** Crystallographic structure of tri-layer Hg1223. We sketch the presence of AFM order in the IP (purple arrow) and charge order (orange wave) in the OPs. **b** Corresponding reconstructed Fermi surface in presence of AFM order in the IP leading to a hole pocket (purple, *F*_2_ QO frequency) and CO order in the OP leading to an electron pocket (orange, *F*_3_ QO frequency). Both pockets are located in the nodal region of the quarter of the first Brillouin zone. Magnetic breakdown tunnelling between the pockets leads to an additional frequency *F*_1_ ≈ *F*_3_ − *F*_2_.

Given the charge imbalance between IP and OP, the carrier density is always lower in the IP. Let us assume that an AFM metallic phase is present in the IP in analogy with the O223F compound^4,34^. But compared to scenario (3), the FS in the IP consists solely of hole pockets at the nodes corresponding to *F*_*hole*_ = *F*_2_ ≈ 500 T. This is in agreement with recent ARPES and QOs studies showing the metallic character of the AFM phase at low doping in the IPs of a 5-layer cuprate^5^. Kunisada et al. found two QO frequencies *F*(IP0) = 147 T and *F*(IP1) = 318 T corresponding to an effective carrier density *p* = 2.1% and *p* = 4.5%, respectively. In our study, the hole frequency *F*_2_ ≈ 500 T translates to a carrier density *p* = 7.2%, in good agreement with the estimation given by NMR measurements in a 3-layer cuprate with *T*_*c*_ = 76 K, where *p*(IP) ≈ 7.4% and *p*(OP) ≈ 8.7%^4^. In addition, as shown by recent Raman spectroscopy measurements in underdoped Hg1223^8^, we assume that the CO sets in the OP as the effective doping is higher. It induces a Fermi surface reconstruction leading to an electron pocket at the node corresponding to *F*_*electron*_ = *F*_3_ ≈ 850 T, in analogy with the monolayer Hg1201. In Fig. 4a, we sketch the real space structure corresponding to this scenario: an AFM order in the IP coexisting with a charge order in the OPs. Figure 4b shows the resulting Fermi surface consisting of both electron (orange) and hole (purple) pockets. The third frequency *F*_*MB*_ = *F*_*e*_ − *F*_*h*_ would correspond to magnetic breakdown tunnelling between OP and IP. Note that the amplitude of the different frequencies depends on the value of *t*_⊥_ and the broadening of the Landau level due to disorder^31^. But how can we reconcile this scenario with a positive Hall effect? Let us focus on the low-temperature value of the Hall coefficient and assume the low-field limit for the two-band model of the Hall effect (see Supplementary Note 5). The Hall coefficient is given by: ${R}_{H}=\frac{{\sigma }_{h}{\mu }_{h}-{\sigma }_{e}{\mu }_{e}}{{({\sigma }_{h}+{\sigma }_{e})}^{2}}$, where *σ* and *μ* are the conductivities and mobilities, respectively. Given the carrier densities deduced from quantum oscillation frequencies, a Hall coefficient *R*_*H*_ ≈ 6.5 mm^3^/C (see Fig. 3) corresponds to a ratio of mobilities *μ*_*h*_/*μ*_*e*_ ≈ 3, a reasonable value owing to the disorder-protected nature of the IP compared to the OPs. In short, this interpretation allows explaining both the QO spectrum and the value of *R*_*H*_ at low temperatures. We cannot exclude that the strong magnetic field influences the ground state of Hg1223 as it is the case for the 3D charge order in underdoped YBCO^19^. However, this is clearly not the case in the 5-layer cuprates, where both ARPES at zero fields and QOs at high field give the same Fermi surface for the two inequivalent inner planes^5^.

Our interpretation based on scenario (4) implies that, in the cuprate where *T*_*c*_ is maximum among all superconductors, a metallic AFM state extends deep inside the SC phase. This is reminiscent of a quantum critical point scenario observed in other unconventional superconductors, where spin fluctuations extend away from the AFM ordered state. The dispersion of such magnetic excitations has first been measured in YBCO using inelastic neutron scattering^35^. Resonant inelastic x-ray scattering (RIXS) experiments have subsequently extracted the dispersion of these magnetic excitations, called paramagnons, up to high energy transfer in different cuprate families and over a large doping range^36^. Interestingly, a recent RIXS study on the two first members of the Hg-family of cuprates shows that the energy scale of the paramagnon spectra matches the ratio of *T*_*c*_^37^. All of the above considerations strongly suggest a magnetic pairing mechanism for cuprates. In Hg1223, the clean nature and the absence of buckling of the inner CuO_2_ plane support the idea that the antiferromagnetic interaction *J* is large, leading to higher *T*_*c*_^25,37^. Could the presence of charge order in the OPs be a consequence of charge imbalance and / or of out-of-plane disorder? In YBCO, charge order competes both with SC and AFM order. This could explain why *T*_*c*_ further increases in optimally doped Hg1223 by applying pressure^38^ which destabilizes charge order^39^.

## Methods

### Samples

Single crystals of the tri-layer cuprate HgBa_2_Ca_2_Cu_3_O_8+*δ*_ have been synthesized using a self-flux growth technique as described in ref. ^10^. Using adequate heat treatment, Hg1223 can be largely underdoped and its doping level controlled. The doping *p* has been deduced from the empirical relation 1 − *T*_*c*_/*T*_*c*,*max*_ = 82.6(*p* − 0.16)^2^, where *T*_*c*_ is the onset superconducting transition measured by SQUID (see Supplementary Fig. 1) and *T*_*c*,*max*_ = 133 K.

### TDO measurements

Quantum oscillations have been measured using a contactless tunnel diode oscillator-based technique^40^ in two samples of Hg1223 at doping level *p* = 8% and *p* = 8.7%. Typical sample dimensions are 500 × 500 × 100 μm^3^. The experimental setup consists of a LC-tank circuit powered by a tunnelling diode oscillator biased in the negative resistance region of the current-voltage characteristic. The sample is placed in a compensated 8-shape coil (diameter and length of the coil are adapted for each sample to optimize the filling factor). The fundamental resonant frequency *f*_0_ of the whole circuit is around 25 MHz. The RF signal is amplified and demodulated down to a frequency of about 1 MHz using a heterodyne circuit. A high-speed acquisition system is used to digitize the signal. The data are post-analyzed using software to extract the field dependence of the resonance frequency *f*_*TDO*_, which is sensitive to the resistivity through the change in skin depth.

### Hall effect measurements

The Hall effect was measured in the third sample of Hg1223 at doping level *p* = 8.8%. The dimensions of the sample are 700 × 450 × 90 μm^3^. Gold contacts were sputtered onto the surface of the sample before a heat treatment leading to contact resistances of a few ohms at room temperature and below 1 Ω at low temperature. The magnetic field *H* was applied along the *c*-axis of the tetragonal structure, perpendicular to the CuO_2_ planes in both polarities of the field. The high-temperature measurements were performed in a conventional pulsed magnet up to 68 T down to 10 K. At lower temperature, higher magnetic fields were required to quench superconductivity, so we extended our measurements up to 88 T, using a dual coil magnet. The pulsed-field measurements were performed using a conventional 4-point configuration with a current excitation of 5 mA at a frequency of 60 kHz. A high-speed acquisition system was used to digitize the reference signal (current) and the voltage drop across the sample at a frequency of 500 kHz. The data were post-analyzed with software to perform the phase comparison.

## Supplementary information

Supplementary Information

## Data Availability

The data that support the findings of this study are available from the corresponding authors upon reasonable request.
