# Supplementary material for: Magnetotransport signatures of antiferromagnetism coexisting with charge order in the trilayer cuprate HgBa2Ca2Cu3O8+δ
Source: Nat Commun. 2022 Mar 23;13:1568. doi: 10.1038/s41467-022-29134-6 (PMC8943046; doi:10.1038/s41467-022-29134-6)
Supplement: Supplementary file 1 — Supplementary Information [file 41467_2022_29134_MOESM1_ESM.pdf]

SUPPLEMENTARY INFORMATION for
"Magnetotransport signatures of antiferromagnetism coexisting with charge order in the trilayer cuprate $HgBa_2Ca_2Cu_3O_{8+\delta}$"

V. Oliviero, S. Benhabib, I. Gilmutdinov, B. Vignolle, L. Drigo, M. Massoudzadegan, M. Leroux, G.L.J.A. Rikken, A. Forget, D. Colson, D. Vignolles and C. Proust

Supplementary Note 1. Magnetization measurements

Fig. 1 shows the temperature dependence of the magnetic susceptibility of the three samples of $HgBa_2Ca_2Cu_3O_{8+\delta}$. T_c corresponds to the onset temperature where the susceptibility starts to drop. The two samples of Hg1223 at $p = 8$ % ($T_c = 64$ K, black line) and $p = 8.7$ % ($T_c = 74$ K, red line) have been studied by quantum oscillation measurements using the tunnel diode oscillation (TDO) technique. Hall effect measurements have been performed on the third sample of Hg1223 at $p = 8.8$ % sample ($T_c = 78$ K, blue line).

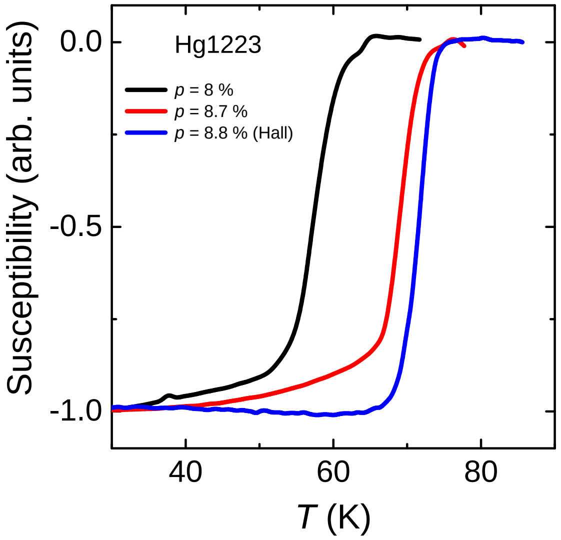

FIG. 1: **SQUID data.** Magnetic susceptibility measurements of vacuum-annealed crystals of $HgBa_2Ca_2Cu_3O_{8+\delta}$ at different doping levels, as indicated.

Supplementary Note 2. Quantum oscillation data

The complete data of quantum oscillations in two samples of Hg1223 is displayed in Fig. 2. Figs. 2a, b show the variation of the Tunnel Diode Oscillator (TDO) frequency as a function of magnetic field up to $H = 88$ T at different temperatures for the samples $p = 8$ % and $p = 8.7$ %, respectively. The thin lines correspond to the smooth background (splines) subtracted from the raw data leaving the oscillatory part of the signal depicted in Fig. 2c ($p = 8$ %) and Fig. 2d ($p = 8.7$ %). Figs. 2e, f show the discrete Fourier transform of the oscillatory part in the field range [35 T, 85.5 T] ($p = 8$ %) and [37 T, 85.3 T] ($p = 8.7$ %), respectively. Table 1 shows the effective masses of the different frequencies obtained from the temperature dependence of the amplitude of the oscillation given by the Fourier transform. It is worth to note that the background of the TDO signal is strongly field dependent. Since the amplitude of the QOs is small compared to this background, its subtraction is not straightforward, in particular in presence of several QO frequencies. The peaks at low frequency in the Fourier transform are artefacts coming from the fact that the oscillatory part of the TDO signal is not perfectly symmetric about the x-axis. Since we have a limited field range to analyse the QOs, a field window H =35 T − 85.5 T corresponds to less than 2 oscillations for a frequency of F = 110 T. We cannot exclude the presence of such low frequency but it is impossible to reveal it by Fourier transform. A strong argument against the presence of such low QO frequency is the temperature dependence of the peak in the Fourier transform, that is not in agreement with the behaviour expected for QO.

Family	T_c	p	m^*_{c1}	m^*_{c2}	m^*_{c3}
Hg1223	64 K	8.0 %	0.75±0.2	1.0±0.2	1.5±0.2
Hg1223	74 K	8.7 %	N/A	2.2±0.3	2.4±0.2

TABLE 1: Effective masses deduced from the Fourier transform analysis for the Hg1223 samples.

Supplementary Note 3. Fitting quantum oscillations

In order to confirm the value of the frequencies obtained by Fourier transform, some fits of the QOs were performed. This is an additional check of our analysis leading to our

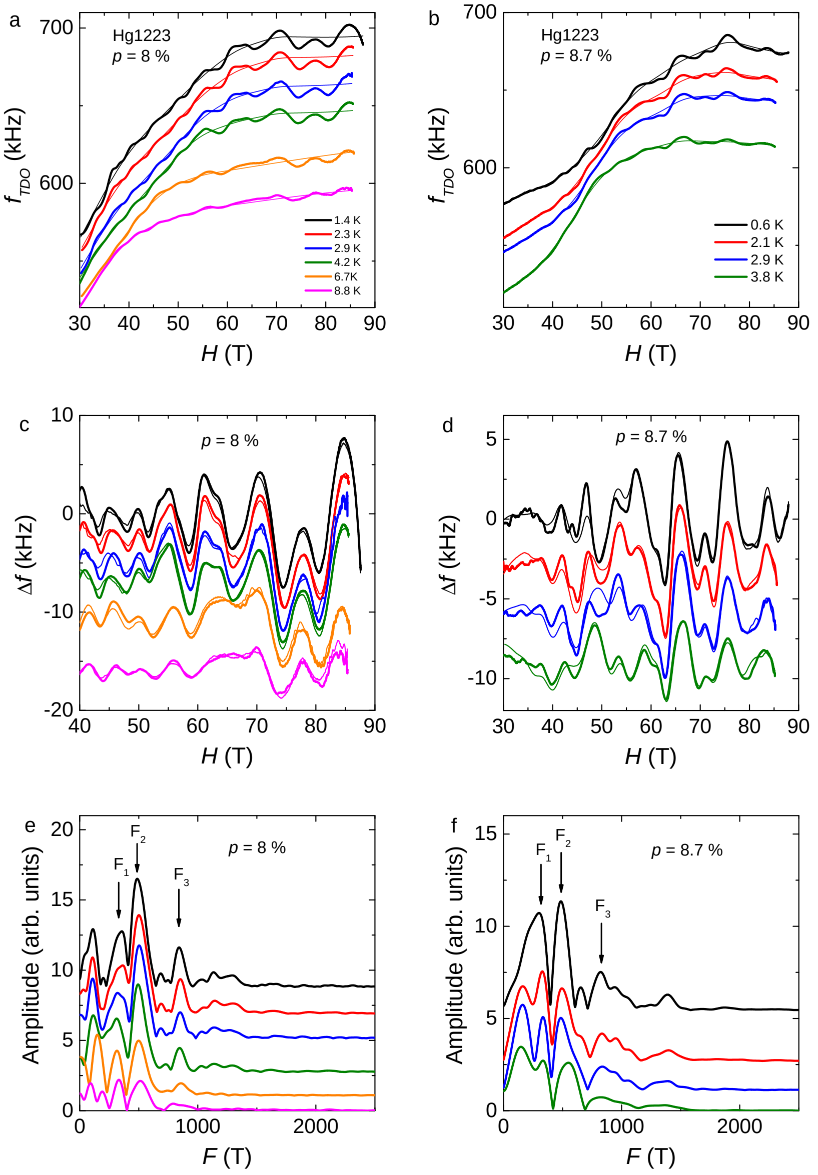

FIG. 2: **TDO data.** a) Field dependence of the TDO frequency after the heterodyne circuit at low temperature in the trilayer Hg1223 $p = 8$ % at different temperatures, as indicated. The thin lines correspond to the background (spline) of the TDO signal. b) Same graph but for our Hg1223 $p = 8.7$ % sample. Oscillatory part of the TDO signal after removing a smooth background (spline) for c) $p = 8$ % and d) $p = 8.7$ %. The thin (thick) lines correspond to the rising (falling) of the magnetic field. e) Discrete Fourier analysis in the field range $H = 35$ - 85.5 T of the data shown in panel c) for $p = 8$ %. Arrows indicate the three frequencies discussed in the main. f) Discrete Fourier analysis in the field range $H = 37$ - 85.3 T of the data shown in panel d) for $p = 8.7$ %. Arrows indicate the three frequencies discussed in the main.

interpretation. We fit the field dependence of the TDO signal to $f_{TDO} = (a_0 + a_1 H + a_2 H^2 + ...) + \Delta f_{osc}$, where the first term is a polynomial representing the non-oscillating background and Δf_{osc} is given by the Lifshitz-Kosevich (LK) theory,

$$\Delta f_{osc} = \sum_i A_{0i} R_{Ti} R_{Di} sin[2\pi(\frac{F_i}{H} - \gamma_i)] \quad (1)$$

where A_{0i} are prefactors, F_i are the oscillation frequencies and γ_i are the phase factors. We neglect any contribution from magnetic breakdown and spin damping. R_{Ti} and R_{Di} are the thermal ($R_{Ti} = \alpha T m_i^*/H sinh[\alpha T m_i^*/H]$) and Dingle ($R_{Di} = exp[-\alpha T_{Di} m_i{}^*/H]$) damping factors, respectively, where $\alpha = 2\pi^2 k_B m_0/e\hbar$ ($\simeq$ 14.69 T/K), m_i^* and T_{Di} are the cyclotron masses and the Dingle temperatures, respectively [1]. Some of the scattering going into T_D probably comes from scattering due to vortices, but we leave this as a field-independent scattering contribution for simplicity. For each frequency, there are five free parameters. In order to constrain the parameters, we perform simultaneous fits with equation (1) to the total data set at different temperatures, where all parameters are temperature independent. We use a gradient based search algorithm where the optimal solution is the set of parameters that gives the smallest least squares value. The gradient based search algorithm we used converges only in the temperature range from T = 1.4 K to T = 4.2 K at p = 8 % and from T = 0.6 K to T = 2.9 K at p = 8.7 %. Due to local minima, there exist a large number of solutions and we have constrained a few parameters like the effective masses (to agree with the Fourier transform analysis) and the Dingle temperature (to take into account the disorder protected nature of the inner plane). Fig. 2 of the main text shows the raw data (symbols) for the p = 8 % sample along with the results of the fitting procedure (black lines) at different temperatures from T=1.4 K to T=4.2 K and in the field range H = 40 - 83 T. Table 2 shows the relevant parameters deduced from the fit, e.g. frequencies, effective masses and Dingle temperatures. The contribution of each frequency to the total signal is seen in the DFT depicted in the inset of Fig. 2. In addition to the fundamental frequencies, F_1 = 331 T (blue), F_2 = 500 T (grey) and F_3 = 866 T (red), a harmonic of the signal at F_4 = 1150 T has been taken into account to improve the fit. The simulated TDO signal does not reproduce perfectly the data due to a complicated background and additional harmonics of the signal. Nevertheless, the oscillation frequencies match the value obtained by the DFT within the error bars (see table I of the main text).

The same procedure was carried out for the p = 8.7 % sample in the temperature range

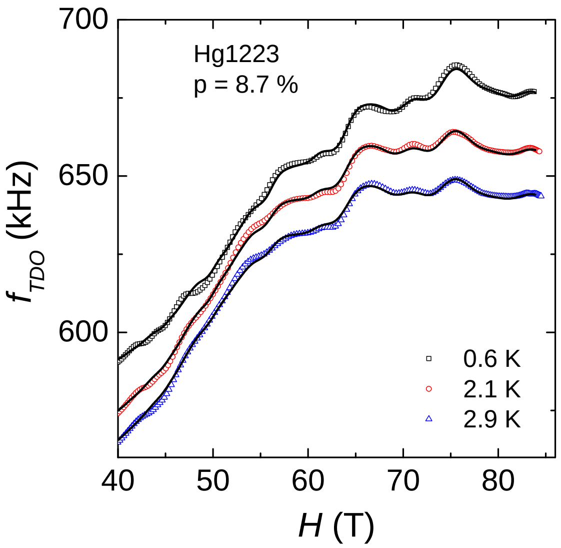

FIG. 3: **Lifshitz-Kosevich fit.** Field dependence of the TDO frequency in Hg1223 ($p =$ 8.7 %) at different temperatures (symbols). Solid lines correspond to the fit to the data using the Lifshitz-Kosevich theory plus a polynomial background in the field range $40 \leq H \leq 85$ T (see text).

	$p = 8$ %			$p = 8.7$ %		
	F_1	F_2	F_3	F_1	F_2	F_3
F (T)	331	500	866	333	514	820
m_c^* (m_e)	1.9	1.2	1.6	1.6	2	2.2
T_D (K)	8	7	14	5	7	16

TABLE 2: QO frequencies, effective masses and Dingle temperatures deduced from the fit of the TDO signal to the Lifshitz-Kosevich theory (see text) for the $p = 8$ % sample (see Fig. 2) and the $p = 8.7$ % sample (see Fig. 3).

T=0.6 K to T=2.9 K and in the field range H = 40 - 85 T (see Fig. 3). Table 2 shows the relevant parameters deduced from the fit, i.e. frequencies, effective masses and Dingle temperatures. Again, the oscillation frequencies match the values obtained by the DFT within the error bars (see table 1 of the main text).

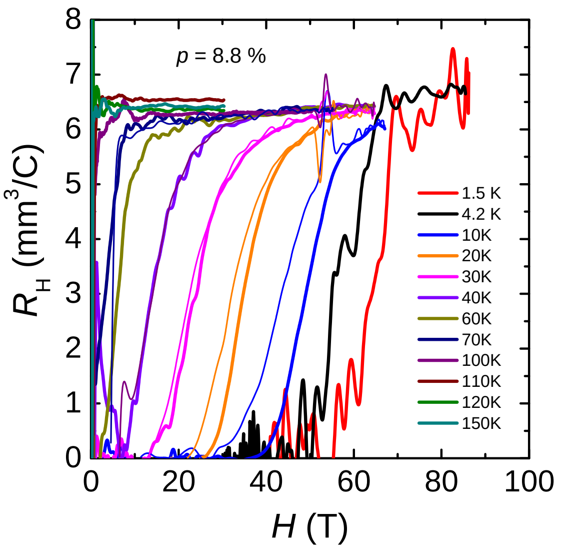

FIG. 4: **Field dependence of the Hall coefficient in Hg1223.** Hall coefficient (R_H) of Hg1223 ($p = 8.8$ %) at various fixed temperatures, as indicated, versus magnetic field. The thin (thick) lines correspond to the rising (falling) of the magnetic field for the measurements up to 68 T. Data for the rising of the magnetic field in the 88 T coil are too noisy.

Supplementary Note 4. Hall effect data

The transverse Hall resistance R_{xy} of our Hg1223 $p = 8.8$ % sample was measured in pulsed fields. Fig. 4 shows the isotherms of the Hall coefficient, $R_H = tR_{xy}/H$, as indicated, where t is the thickness of the sample. Data in the temperature range $T = 10$ - 150 K was obtained in a 68 T pulsed magnet, where the signal to noise ratio was good. However, at $T = 10$ K, 68 T was not enough to reach the normal state value of the Hall coefficient. We have thus performed additional measurements in a dual coil magnet to produce non-destructive magnetic fields up to 88 T at lower temperature, $T = 4.2$ K and $T = 1.5$ K. Due to larger $\mathrm{d}H/\mathrm{d}t$, the signal to noise ratio is worse but we can clearly state that R_H remains positive down to the lowest temperature. This means that the Fermi surface of Hg1223 contains at least one mobile hole pocket at this doping level (see next section).

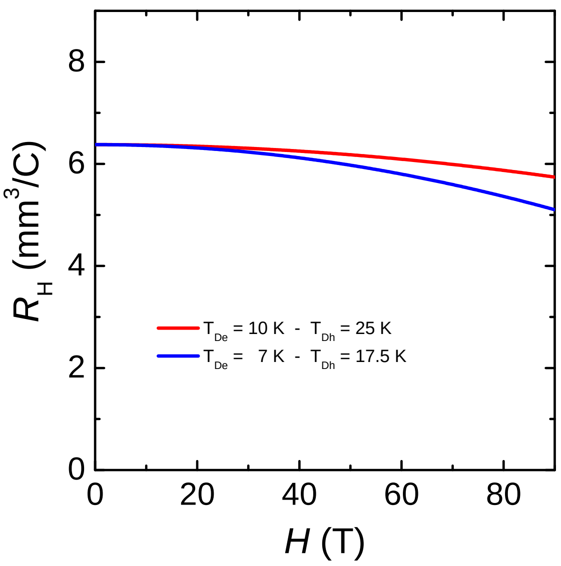

FIG. 5: **Field dependence of the Hall coefficient in the two-band model.** Simulated R_H versus magnetic field in the two-band model (see text). We use $m_{ce} = 2.5\ m_0$ and $m_{ch} = 2.2\ m_0$ for the effective mass of the electron (outer planes) and the hole (inner plane), respectively. The Dingle temperatures of the electron and the hole are indicated.

Supplementary Note 5. Two-band model

In the main text, we argue that one scenario compatible with the QO spectrum consists of antiferromagnetism (hole pocket) in the inner plane and charge order (electron pocket) in the outer plane (see Fig. 4b of the main text). Since the inner plane is protected from out-of-plane disorder, we assume that the hole-like carriers are more mobile than the electron-like carriers.

Using a simple Drude model in the low field limit, the Hall coefficient is given by:

$$R_H = \frac{\sigma_h \mu_h - \sigma_e \mu_e}{(\sigma_h + \sigma_e)^2} \tag{2}$$

where σ and μ are the conductivities and mobilities, respectively. To calculate the carrier density, we rely on the measured QO frequencies. According to Onsager's relation, the size of the Fermi surface pocket A_{FS} in reciprocal space is :

$$A_{\mathrm{FS}} = \frac{2\pi e F}{\hbar}, \tag{3}$$

where F is the SdH oscillation frequency. The number of carriers per layer is given by:

$$N = \frac{2A_{\mathrm{FS}}}{A_{\mathrm{FBZ}}}, \tag{4}$$

where $A_{\rm FBZ}$ is the size of the first Brillouin zone in the reciprocal space. In a unit cell there are three layers with one Cu atom per layer. We suppose that the Fermi surface of the outer planes contains a single electron pocket due Fermi surface reconstruction by charge order. The Fermi surface of the inner layer contains two hole pockets due to Fermi surface reconstruction by antiferromagnetism. Then the carrier density $n_{h(el)}$ of holes(electrons) is calculated according to the following formula:

$$n_{\rm h} = \frac{N_{\rm h}}{V}, n_{\rm el} = \frac{2N_{\rm el}}{V} \tag{5}$$

where $V = 236.9$ $\mathring{\rm A}^3$ is the unit cell volume of Hg1223. As stated in the main text, a Hall coefficient $R_H \approx 6.5$ mm^3/C (see Fig. 3) corresponds to a ratio of mobilities μ_h/μ_e =2.85, a reasonable value owing to the disorder-protected nature of inner plane compare to outer plane. Now we have to justify the low-field limit. This is in fact indicated in the data of Fig. 4 by the slight field dependence of R_H. In the field-dependent two-band model, R_H is given by:

$$R_H(H) = \frac{\sigma_h^2 R_h + \sigma_e^2 R_e + \sigma_h^2 \sigma_e^2 R_h R_e (R_h + R_e) H^2}{(\sigma_h + \sigma_e)^2 + \sigma_h^2 \sigma_e^2 (R_h + R_e)^2 H^2} \tag{6}$$

where $\sigma_{h(e)}$ and $R_{h(e)}$ are the conductivity and Hall number of the $h(e)$ carriers, respectively: $\sigma_{h(e)} = en_{h(e)}\mu_{h(e)}$ and $R_{h(e)} = \frac{1}{en_{h(e)}}$.

Fig. 5 shows the simulated Hall coefficient using a set of parameters compatible with the effective masses within error bars (see Table 1) and the order of magnitude of the Dingle temperature for the $p = 8.7$ % sample (see Table 2). We use the parameters of the $p = 8.7$ % sample since Hall effect measurements have been performed in a sample with a similar doping level, namely $p = 8.8$ %. As seen in Fig. 5, we expect a slight departure from the low field limit since the Hall coefficient is not absolutely constant over our field range. Due to the signal-to-noise ratio at low temperature and the limited field range, it is hard to tell from the experimental data if such departure of the low field limit exists. Note that this model is very simple since for instance, it does not take into account the anisotropy of the scattering rate along the Fermi surface, which can be substantial in the cuprates. But one should keep in mind that the main target of the analysis is to get reliable value of the QO frequencies and explain the positive Hall number at low temperature.

Supplementary Note 6. Sketch of Fermi surface

The sketches presented in Fig. 4b of the main text and Fig. 6 are based on calculations of Fermi surface reconstruction due to AFM order and/or CDW order. For the sake of simplicity, we treat the inner and the outer planes as independent even if they are coupled by an interlayer tunneling $t_\perp$. For the Fermi surface reconstruction, we consider only one band within each CuO_2 plane. The starting point of the calculations is the unreconstructed Fermi surface of the single-layer Hg1201 compound, parametrized by the tight-binding equation:

$$E(k) = -2t_1(cos(ak_x) + cos(bk_y)) - 4t_2(cos(ak_x) \times cos(bk_y)) - 2t_3(cos(2ak_x) + cos(2bk_y)) \\ - 4t_4(cos(2ak_x) \times cos(bk_y) + cos(ak_x) \times cos(2bk_y)) - \mu \quad (7)$$

where $(t_1, t_2, t_3, t_4) = (0.48, -0.105, 0.08, -0.02)$ eV, k_x and k_y are the in-plane wavevectors, a and b the in-plane lattice constants. The tight-binding parameters used in the calculations are chosen in order to reproduce the photoemission data reported in Hg1201 [2]. The chemical potential (μ) is adjusted to yield a doping level p = 8.5% (for μ = -0.355 eV) so that the total carrier density is equal to 1 + p holes, according to the Luttinger sum rule.

For the calculation of Fermi surface reconstruction due to AFM order, we used the following analytical formula for the electron ($E_e(k)$) and hole ($E_h(k)$) band structure:

$$E_e(k) = \frac{E(k) + E(k + Q_{AF})}{2} + \sqrt{\frac{(E(k) - E(k + Q_{AF}))^2}{4} + V_{AF}^2} \quad (8)$$

$$E_h(k) = \frac{E(k) + E(k + Q_{AF})}{2} - \sqrt{\frac{(E(k) - E(k + Q_{AF}))^2}{4} + V_{AF}^2} \quad (9)$$

where V_{AF} is the AFM potential, $Q_{AF} = (\pi, \pi)$ is the antiferromagnetic wavevector. The hole pocket (purple FS in Fig. 4) whose area corresponds to a frequency of 500 T is obtained using $V_{AF} = 0.445$ eV.

For the calculation of Fermi surface reconstruction due to bi-axial charge order, only the first-order translations of the band-structure due to charge-ordering with a characteristic wavevector Q_{CDW} are considered [3]. The resulting 4×4 Hamiltonian is then diagonalized numerically, with a typical mesh of 10^6 points. We choose $Q_{CDW} = 0.275$ r.l.u and

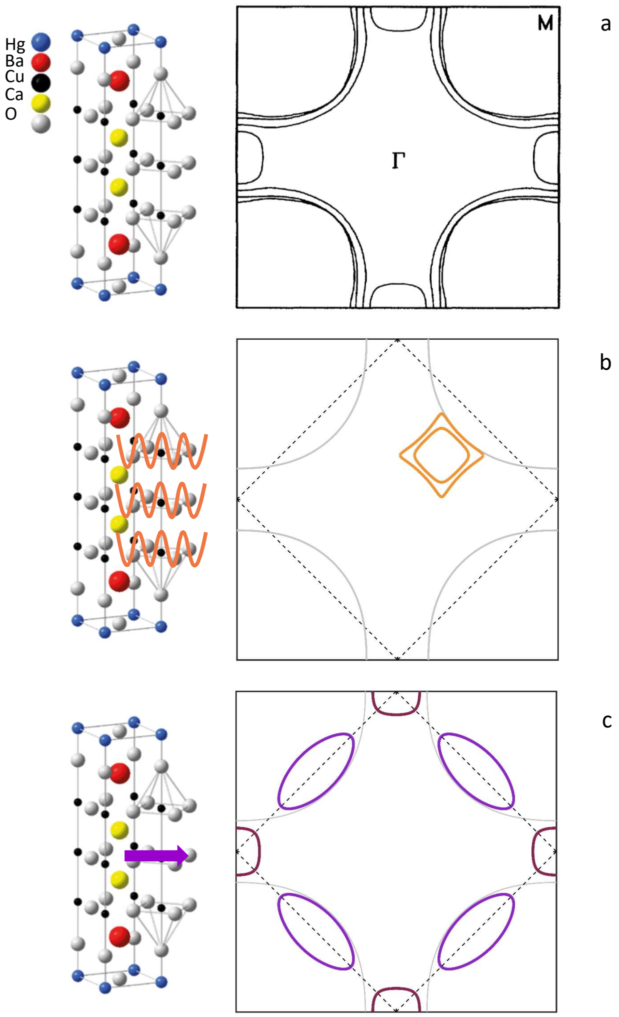

FIG. 6: **Sketch of the Fermi surface.** To illustrate the discussion in the main text regarding the different scenarios, we show a) FS obtained by LDA calculation (from Ref. 4). b) Sketch of the FS assuming a Fermi surface reconstruction by charge order in the three CuO_2 planes. c) Fermi surface of the inner plane assuming a Fermi surface reconstruction by antiferromagnetism.

$V_{CDW} = 0.075$ eV to produce the electron pocket with an associated frequency of 850 T displayed as an orange line in Fig. 4 of the main text.

Fig. 6a reproduces the Fermi surface of the stoichiometric compound $HgBa_2Ca_2Cu_3O_8$ obtained by local density approximation calculations [4]. It consists of three large hole-like tubular CuO_2 sheets centered on the corner of the Brillouin zone plus a small electron-like

Fermi surface located at the anti-node. The latter disappears with doping [4]. The size of the large orbits translates into a QO frequency of about 15 kT, a value much larger than the observed frequencies.

Fig. 6b corresponds to the Fermi surface of Hg1223 assuming a Fermi surface reconstruction by charge order in the three CuO_2 planes. Here, we assume that the Fermi surfaces derived from the outer planes are similar in size. Therefore, a Fermi surface reconstruction by charge order leads to two electron pockets located at the node. For the sake of simplicity, solely the strength of the CDW potential V_{CDW} was adjusted to produce the electron pockets with F = 850 T and F = 500 T displayed in figure 6b. Similar results could be obtained by varying the CDW wavevector Q_{CDW}.

Fig. 6c depicts the Fermi surface of the inner plane assuming a Fermi surface reconstruction by AFM order. The hole (electron) FS presented as purple (wine) lines in Fig. 6c corresponding to a quantum oscillation frequency of $F_h = 850$ T ($F_e = 500$T) are obtained using $V_{AF} = 0.25$ eV. They are compatible with the observed QO frequencies F_2 and F_3. The third observed frequency ($F_1 = 350$ T) could then be due to magnetic breakdown between these two orbits. However, the magnetic breakdown field B_0 required to enable magnetic breakdown orbits is $B_0 \propto \Delta k^2$ where Δk is the separation between the two orbits. This large gap precludes the observation of magnetic breakdown in the QOs. A simple estimate of the magnetic breakdown field can be achieved using the Blount criterion:

$$B_0 = \frac{m^*}{e\hbar} \times \frac{E_g^2}{E_F} \tag{10}$$

where $E_F = \frac{e\hbar}{m^*} \times F_{QO}$ is the Fermi energy, E_g is the energy gap due to AFM order, m^* the effective mass and F_{QO} the QO frequency. Using $E_g = 0.25$ eV, $m^* = 1.6m_0$ ($1.2m_0$) and $F_{QO} = 850$ T (500 T) yields an estimated magnetic breakdown field $B_0 \approx 13000$ T (14000T), more than two orders of magnitude larger than the magnetic field used in the present study.

Supplementary References

[1] D. Shoenberg, *Magnetic Oscillations in Metals* (Cambridge University Press, Cambridge, 1984).

[2] Vishik, I.M. *et al.* Angle-resolved photoemission spectroscopy study of $HgBa_2CuO_{4+\delta}$. *Phys. Rev. B* **89**, 195141 (2014).

[3] Harrison, N. and Sebastian, S.E. Protected Nodal Electron Pocket from Multiple-Q Ordering in Underdoped High Temperature Superconductors. *Phys. Rev. Lett.* **106**, 226402 (2011).

[4] Singh, D.J. Electronic structure of $HgBa_2Ca_2Cu_3O_8$: The role of mercury. *Phys. Rev. B* **48**, 3571 (1993).
